# Meta-analysis of the risk of cataract in type 2 diabetes

**DOI:** 10.1186/1471-2415-14-94

**Published:** 2014-07-24

**Authors:** Li Li, Xiu-hua Wan, Guo-hong Zhao

**Affiliations:** 1 Department of Ophthalmology, The Affiliated Beijing Children’s Hospital of Capital Medical University; National Key Discipline of Pediatrics, Ministry of Education, Beijing 100045, China; 2 Beijing TongRen Eye Center, Beijing TongRen Hospital, Capital Medical University; Beijing Ophthalmology and Visual Sciences Key Laboratory, Beijing 100730, China

**Keywords:** Non-diabetic, Nuclear sclerosis, Ophthalmology

## Abstract

**Background:**

This meta-analysis aimed to investigate the association between type 2 diabetes (T2D) and the risk of cataract.

**Methods:**

Databases of Pubmed, Embase, and SpringerLink were retrieved for observational studies published before November 2013. The odds ratio (OR) and 95% confidence interval (CI) were used for estimating the association. All statistical analyses were performed by Stata 10.0 software.

**Results:**

A total of 8 studies involving 20837 subjects were included in the meta-analysis. The risk of any cataract (AC) in T2D patients was higher than that in non-diabetic subjects (OR = 1.97, 95% CI: 1.45-2.67, *P* < 0.001). The risks of cortical cataract posterior (CC) (OR = 1.68, 95% CI: 1.47-1.91, *P* < 0.001) and posterior subcapsular (PSC) (OR = 1.55, 95% CI: 1.27-1.90, *P* < 0.001) were significantly elevated in T2D patients, while no significant association was found in nuclear sclerosis (NS) (OR = 1.36, 95% CI: 0.97-1.90, *P* = 0.070).

**Conclusion:**

T2D patients had a higher risk of cataracts, excepting NS. Special attention should be paid on the ophthalmic extermination, especially for cataract in T2D patients.

## Background

Cataract, a loss of the normal transparency of the crystalline lens due to an opacity (lens opacity or crystalline opacity), is one of the leading causes of blindness worldwide [1,2]. Hence, identification of the risk factors is of great importance for prevention and treatment of the blindness. Pollreisz [3] propose in a review article that diabetes is one of the widely perceived risk factors for cataract. Diabetes patients are more prone to develop cataracts [2]. The cataract incidence was estimated 3.31 per 1000 person-years of type 2 diabetic patients during 3.6 years’ follow-up [4]. However, studies [5-8] found that not all types of cataracts [9], nuclear sclerosis (NS), cortical cataract (CC) or posterior subcapsular (PSC), are more prone to occurring in type 2 diabetes (T2D) patients. Evidence for their association has not been systematically assessed.

Therefore, we performed this meta-analysis to explore the association between T2D and the risk of cataract. We anticipate the findings of this study will provide reliable evidence for clinical cataract research and prevention.

## Methods

### Search strategy

The databases included PubMed, Embase and SpringerLink and the studies had to be published before November 2013. Only the articles written in English were screened. The key words were consisted of three parts: 1) cataract OR lens opacity OR crystalline opacity; 2) diabetes OR T2DM OR type 2 diabetes; 3) risk OR incidence.

### The eligible criteria

Inclusion criteria were: (1) the study was designed as observational study (cross-sectional, case–control or cohort study); (2) the study explored the relationship between T2D and the risk of cataracts; (3) there was control group; (4) the outcomes include incidence of cataracts (AC, CC, NS and PSC); (5) the study provided enough information for calculating the Odds Ratio (OR) and 95% confidence interval (CI); (6) if there were multiple articles with same population or data, only the article with the longest follow-up and complete data was selected.

Exclusion criteria were: (1) the study with type 1 diabetes mellitus patients was excluded; (2) all duplicates were excluded; and (3) review articles, letters and comments were also excluded.

### Study selection and quality assessment

Two investigators independently retrieved the eligible studies according to the search strategy and eligible criteria. The references were managed by Endnote software (Thomson ISI ResearchSoft, Carlsbad, CA, USA). Besides, the manual search was performed to retrieve some more eligible studies in the reviews and references of included studies. The quality of the selected studies were assessed by STROBE statement [10] including 22 items.

### Data extraction

Study characteristics, including first author, publication year, study design, country, diagnosis of cataract and diabetes, age/gender of patient, were extracted independently by two researchers. The odds ratios (ORs) and 95% confidence intervals (CIs) of the exposures were extracted. The statistical methods of covariates adjustment were also noted. Any disagreement was resolved by discussion.

### Heterogeneity test

The heterogeneity between studies was evaluated by *Q* test [11] and *I*^*2*^ statistics [12], where, *P* > 0.05 and/or *I*^*2*^ < 50% was considered homogeneity, and a fixed-effect model was used for calculate pooled effect; otherwise, there was significant heterogeneity and random-effect model was used.

### Pooled analysis

The meta-analysis was stratified for different types of cataract definition: AC, CC, NS and PSC. The pooled effect of each exposure on T2D was estimated by the values of ORs and 95% CIs. If the ORs were provided in the publications, they were used for pooled estimate. Otherwise, the ORs were calculated according to the provided data in the articles. All statistical analyses were conducted by Stata 11.0 software.

### Sensitivity analysis and publication bias estimate

The sensitivity analysis was conducted to test the robustness of the results by: 1) only the cross-sectional studies were included; 2) only the studies with Eye examination to confirm the cataract were included. The publication bias was estimated by Begg’s test [13] and Egger’s test [14], using a significance level of *P* < 0.05 to indicate significant asymmetry.

## Results

### Study selection

**Figure 1:**
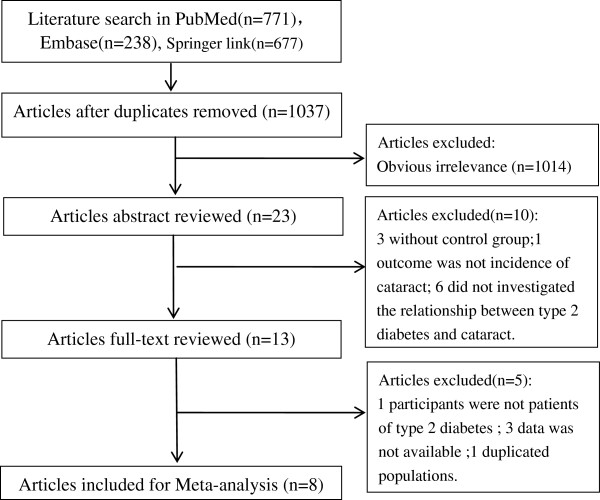
Literature search and study selection.

The process of literature search and study selection was displayed in Figure 1. By retrieval of PubMed, Embase and SpringerLink databases according to the search strategy, 771, 238 and 677 documents were obtained, respectively. After excluding the duplicates, 1037 articles remained. By screening the title, we excluded 1014 documents that did not meet the inclusion criteria. Then by reading the abstracts 10 studies were excluded (3 without control group; 1 outcome was not incidence of cataract; 6 did not investigated the relationship between T2D and cataract). Then in the remaining 13 studies, we reviewed the full text and 5 studies were excluded including 1 with non T2D subjects, 3 with incomplete data and 1 with duplicated crowd. Finally, 8 studies [5-7,15-19] were included in this meta-analysis.

### The characteristics of the included studies

The characteristics of the included studies were listed in Table 1. All studies are with high quality (17–21 STROBE scores, Additional file 1: Table S1). Among the 8 include studies there were 6 cross-sectional studies [5-7,15,17,18], 1 cohort study [19] and 1 case–control study [16], including 20837 subjects. Since Jacques’ study [5] did not provide the specific number of cases, so we could not obtain the accurate total number of cases in this meta-analysis. The area distributions of the 8 studies were: 2 in Europe (France and Sweden), 3 in American, 1 in African and one in Australian. Seven articles reported three kinds of outcomes of NS, CC and PSC. Five studies reported the overall incidence of any cataract (AC). Six literatures provided adjusted OR and 95% CI, two studies provided OR calculable data.

**Table 1:** Characteristics of 8 studies on type 2 diabetes and cataract

**Author year**	**Location**	**Ascertainment of cataract**	**Type of study**	**Ascertainment of diabetes**	**Definition of cataract**	**Age(y) sex**	**Outcome**	**No. of case**	**Diabetes**	**No. of case**	**Non- diabetes**	**ORs (95% CI)**	**Adjustment for covariates**	**STROBE scores**
Machan 2012 [8]	French	Hospital records	Cross- sectional	Hospital records	LOCS II	<1-93 M&F	AC	348	452	1885	5884	1.60 (1.13, 2.27)	Age, gender, smoking, systolic blood pressure, Statin use	20
							NS	282		1546		1.62 (1.14, 2.29)		
							CC	104		525		1.37 (1.02, 1.83)		
							PSC	44		194		1.33 (0.90, 1.96)		
Tan et al. 2008 [19]	Australia	Eye examination	Cohort	Medical record or IFG test	Wisconsin Cataract Grading System	≥49 M&F	NS	37	69	402	1149	6.76 (1.04,14.00)*	Age, gender, smoking, myopia, and pulse pressure, sun-related skin damage, ever use of steroids, myopia, and body mass index	21
							CC	32	95	443	1642	1.60 (0.78, 4.87)*		
							PSC	15	112	162	1844	1.56 (0.72, 3.79)*		
Rotimi et al. 2003 [18]	West African	Eye examination	Cross- sectional	IFG test	—	≥20 M&F	AC	373	831	35	191	3.63 (2.45, 5.37)*	Crude	18
Olafsdottir et al. 2012 [7]	Sweden	Eye examination	Cross- sectional	IFG test	LOCS II score ≥ 2	24-93y M&F	AC	208	275	175	256	1.44 (0.98,2.10)*	Crude	20
							NS	132		131		0.88 (0.63,1.24)*		
							CC	180		131		1.81 (1.28,2.56)*		
							PSC	117		83		1.54 (1.08,2.20)*		
Jacques et al. 2003 [5]	USA	Eye examination	Cross- sectional	IFG test	LOCS III ≥2.5, NS; ≥1.0, CC; ≥0.5,PSC	54-73 F	NS	NR	31	NR	400	1.5 (0.6, 3.5)	Age, smoking , summertime sunlight exposure, and alcohol intake	21
							CC					1.2 (0.6, 2.6)		
							PSC					4.1 (1.8, 9.4)		
Klein et al. 1995 [6]	USA	Eye examination	Cross- sectional	Medical record or IFG test	Wisconsin Cataract Grading System	43-84 M&F	NS	66	384	570	4285	0.93 (0.67,1.29)	Age, gender	17
							CC	81		471		1.72 (1.29,2.30)		
							PSC	19		165		1.09 (0.66,1.78)		
Leske et.al. 1999 [16]	USA	Eye examination	Case- control	Medical record or IFG test	LOCS II grade ≥ 2	40-84 M&F	AC	1800	448	2431	289#	1.85 (1.51, 2.27)	Age, gender	18
							NS		48			1.35 (0.89, 2.05)		
							CC	229	201			1.74 (1.39, 2.18)		
							PSC	851	4			1.88 (0.61, 5.79)		
								17						
Foster et al. 2003 [15]	Singapore	Eye examination	Cross- sectional	Medical record	LOCS III ≥ 4 NS ≥ 2 for CC ≥ 2 for PSC	40-81 M&F	AC	NR	27	NR	1066	2.0 (0.9, 4.5)	Age, gender, body mass index and occupation.	21
							NS					2.8 (0.8, 9.4)		
							CC					3.1 (1.6, 6.1)		
							PSC					2.2 (1.2, 4.1)		

*: ORs was calculated based on literature data; AC: any cataract; NS: nuclear sclerosis; CC: cortical cataract; PSC: posterior subcapsular cataract; NR: not recorded; LOCS, lens opacities classification system #: number of diabetes.

### Meta-analysis of the risk of cataract in T2D patients

**Figure 2:**
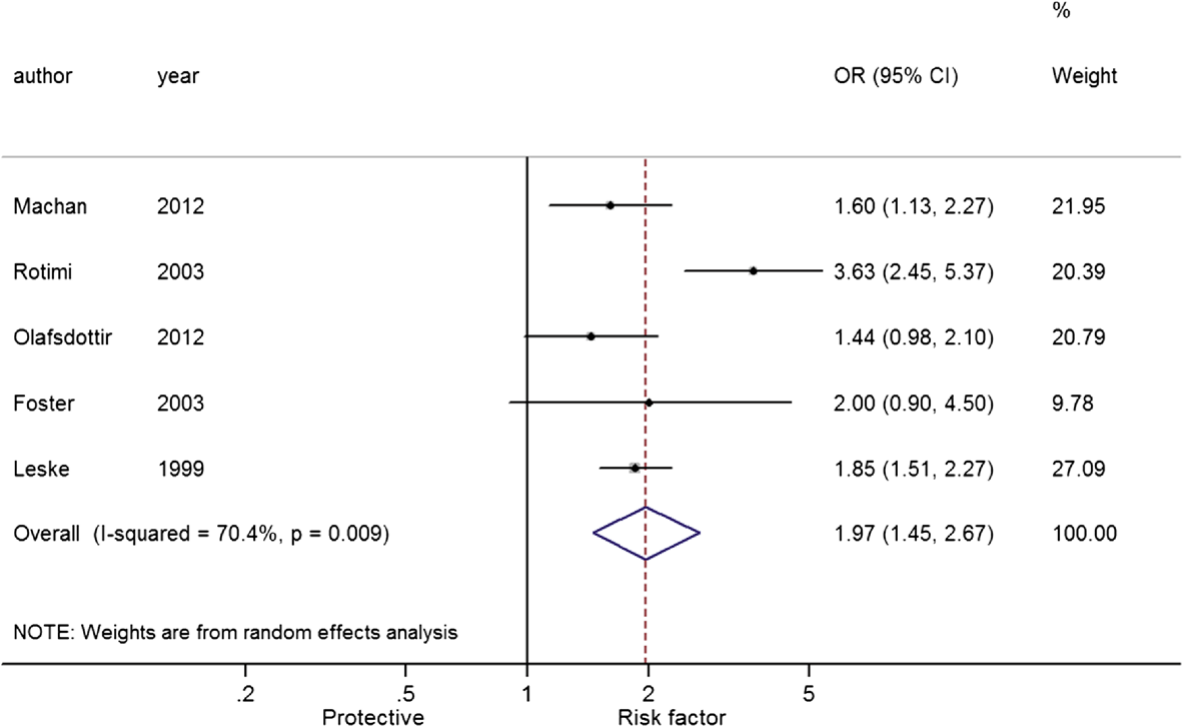
Forest plot of the association between type 2 diabetes and any cataract.

By heterogeneity analysis of the five studies [7,15-18] that reported the overall incidence of AC, there were significant heterogeneity among studies (*I*^2^ = 70.4%, *P* = 0.009), and a random-effect model was used for estimate of the pooled effect. It was showed that (Figure 2) the OR of AC risk between T2D patients and non-diabetic subjects was 1.97 (95% CI: 1.45-2.67, *P* < 0.001), indicating that the risk of AC was significantly elevated in T2D patients compared with non-diabetic subjects.

**Figure 3:**
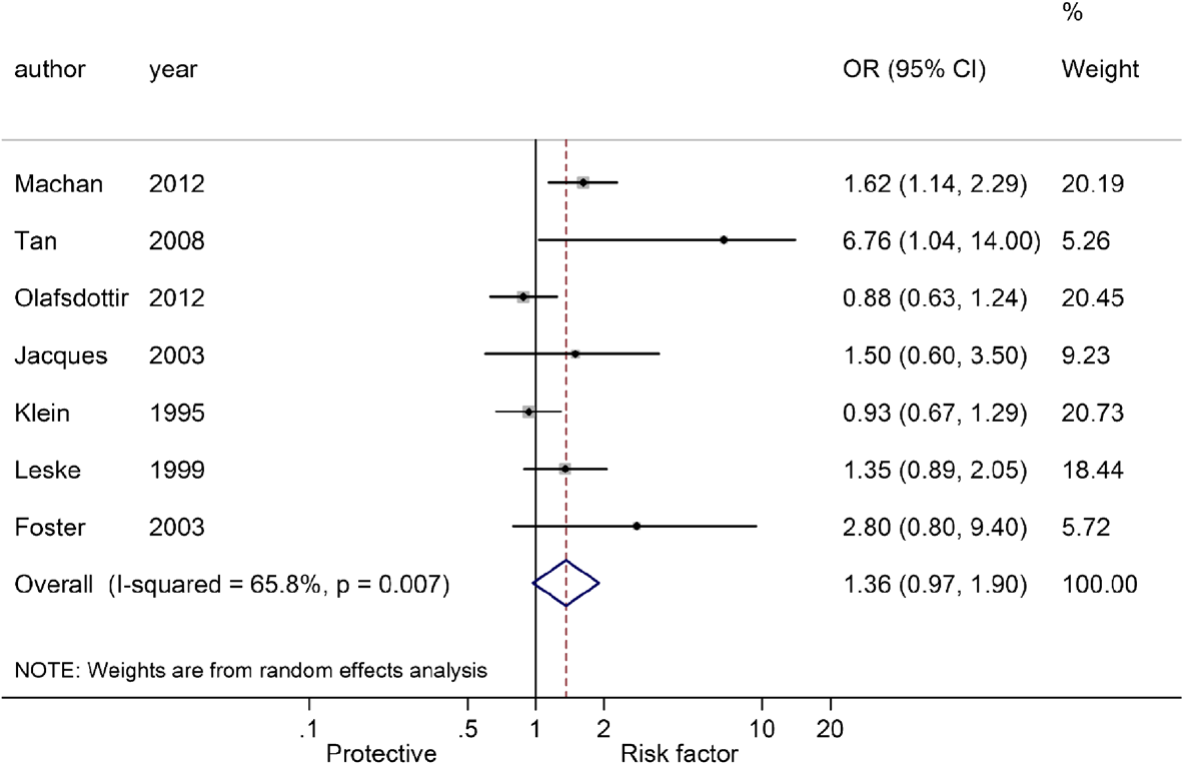
Forest plot of the association between type 2 diabetes and nuclear sclerosis.

**Figure 4:**
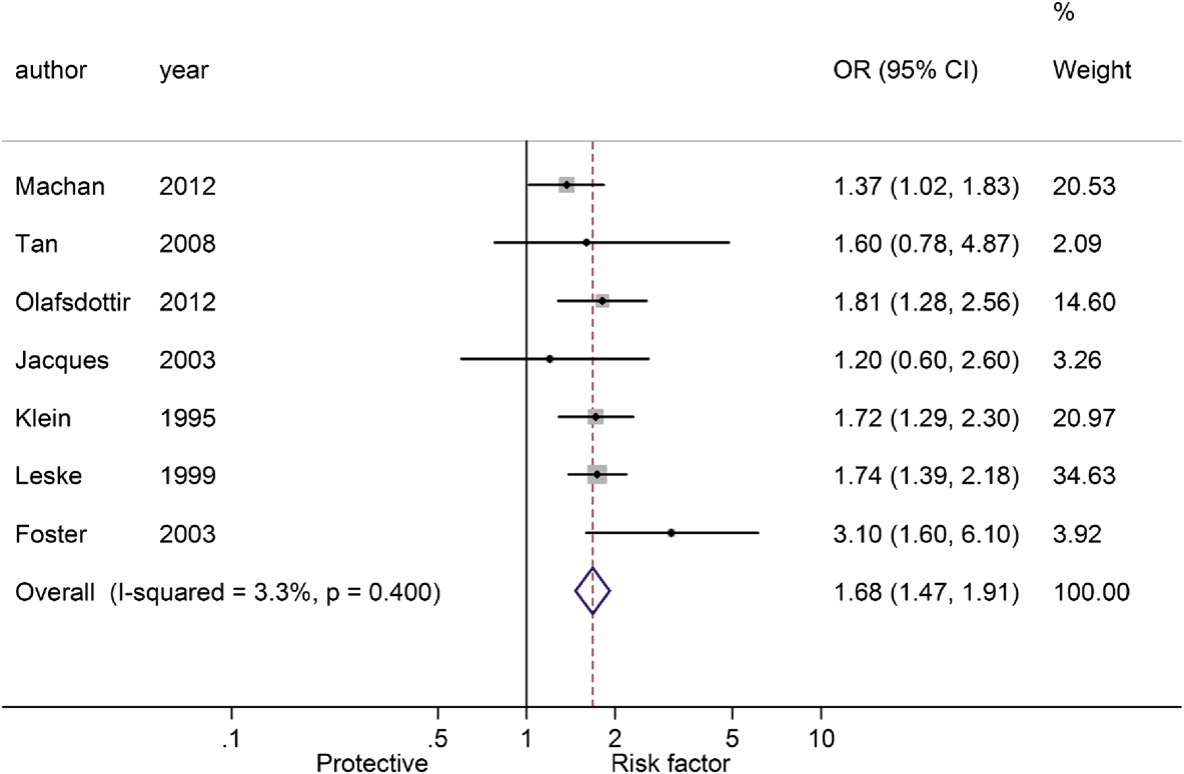
Forest plot of the association between type 2 diabetes and cortical cataract.

**Figure 5:**
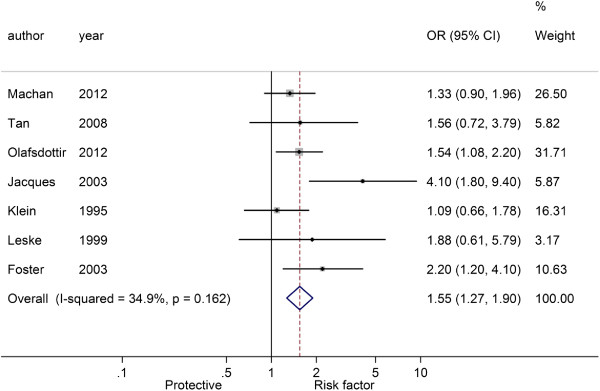
Forest plot of the association between type 2 diabetes and posterior subcapsular cataract.

Figures 3, 4 and 5 showed the pooled results of three types of cataract [5-7,15-17,19], NS, CC and PSC, in T2D patients. There was significant heterogeneity among studies of NS and T2D patients (*I*^2^ = 65.8%, *P* = 0.007), and a random-effect model was used to produce an OR of 1.36 (95% CI: 0.97-1.90, *P* = 0.070), indicating a higher risk of NS in T2D patients over non-T2D patients. There was no significant heterogeneity among studies of CC (*I*^2^ = 3.3%, *P* = 0.400) and PSC (*I*^2^ = 34.9%, *P* = 0.162), and fixed-effect models were used. The pooled ORs were respectively 1.68 for CC (95% CI: 1.47-1.91, *P* < 0.001) and 1.55 for PSC (95% CI: 1.27-1.90, *P* < 0.001). These results indicated that patients with T2D had a higher risk of cataracts than those without.

### Sensitivity analysis and publication bias estimate

The result of sensitivity analysis indicated that the results of the present meta-analysis were robust (Table 2). The pooled results for the outcomes of cross-sectional studies or the studies with Eye examination for cataract diagnosis were consistent with those before sensitivity analysis.

**Table 2:** Sensitivity analysis of meta-analysis of type 2 diabetes and cataract risks

**Outcomes**	**Pooled OR (95%CI)**	***P***_**A**_	***P***_**H**_	**Sensitivity analysis**					
				**Cross-sectional study**	***P***_**A**_	***P***_**H**_	**Eye examination**	***P***_**A**_	***P***_**H**_
Any Cataract	1.97(1.45, 2.67)	<0.001	0.009	2.02(1.27, 3.21)	0.003	0.004	2.09(1.41, 3.09)	<0.001	0.008
NS	1.36(0.97, 1.90)	0.070	0.007	1.20(0.86, 1.69)	0.288	0.040	1.31(0.89, 1.93)	0.164	0.017
CC	1.68(1.47, 1.91)	<0.001	0.400	1.65(1.39, 1.94)	<0.001	0.196	1.77(1.52, 2.05)	<0.001	0.564
PSC	1.55(1.27, 1.90)	<0.001	0.162	1.54(1.25, 1.90)	<0.001	0.059	1.64(1.30, 2.07)	<0.001	0.136

*P*_A_: *P*_value_ of association; *P*_H_: *P*_value_ of Heterogeneity. AC: any cataract; NS: nuclear sclerosis; CC: cortical cataract; PSC: posterior subcapsular cataract.

Begg’s test and Egger’s test showed no significant publish bias among studies (*P* > 0.05, Table 3).

**Table 3:** The results of publication bias estimate

	**Begg’s test**	**Egger’s test**
AC	0.806	0.790
NS	0.368	0.200
CC	1.000	0.906
PSC	0.368	0.198

Data were represented with *P* value.

AC: any cataract; NS: nuclear sclerosis; CC: cortical cataract; PSC: posterior subcapsular cataract.

## Discussion

Cataract is a major cause of blindness worldwide, and it largely results from occurrence of diabetes. The present meta-analysis with a substantial number of subjects (20837 subjects) indicated the risk of cataracts was elevated in T2D patients compared with the non-diabetic subjects.

It was reported that cataract is one of the most common complications of diabetes mellitus on the eye [20,21] and up to 20% of all cataract procedures are performed for diabetic patients [22]. Cataracts were more frequently in patients with diabetes [23,24]. In the present study, approximate 2 times risk of AC was found in T2D patients compared with the non-diabetic subjects. Visual improvement was seen following extracapsular cataract extraction surgery for advanced cataract in diabetics and postoperative monitoring for treatment of diabetic retinopathy may enhance visual outcome [25].

A Waterloo Eye Study by reviewing of 6397 clinic files found that diagnosis of T2D resulted in an earlier development of all three cataract subtypes [8]. Similarly in the present study, we found that the risks of CC and PSC were elevated for patients with the T2D (*P* < 0.05). However, we did not find significant association between T2D and risk of NS. Olafsdottir [7] and Klein [6] reported rather different results about NS from other included studies, which are the main sources of the high heterogeneity, however, they draw similar conclusions in CC and PSC with other included studies. These results highlight the necessary of regular eye examination in T2D patients.

Klein *et al.*[26] indicated that glycemia may be the risk factor of cataracts in T2D patients. Three molecular mechanisms may be involved in the development of diabetic cataract: nonenzymatic glycation of eye lens proteins, oxidative stress, and activated polyol pathway in glucose disposition [27]. In addition, a genetic study showed that three single-nucleotide polymorphisms (SNPs) in chromosome 3p14.1-3p14.2 which related to functions of voltage-dependent anion-selective channel protein, long myosin light chain kinase, adenylyl cyclase-associated protein, and retinoic acid receptor were significantly different in the T2D with cataracts and T2D without cataracts groups [28].

There were limitations in this meta-analysis. Although ORs were corrected by taking account of influences of age, sex and smoking in some included studies, the pooled results might also be influenced by other factors, for instance different treatments of T2D, regions of studies, and body mass index (BMI). Significant heterogeneity still exists among studies, which might be caused by the above factors. In addition, the different methods of definition of cataract (LOCS III, LOCS II, and Wisconsin Cataract Grading System) in deferent studies might also be an important source of heterogeneity.

## Conclusion

In summary, the present meta-analysis of five included studies involving 20837 subjects suggests that T2D is a risk factor of cataract, especially CC and PSC. The findings here attract attentions to the importance of regular ophthalmic extermination in T2D. However, the conclusions need more experimental verification.

## Competing interests

The authors declare that they have no competing interests.

## Authors’ contributions

LL carried out the design and coordinated the study, participated in most of the experiments and prepared the manuscript. XW provide assistance in the design of the study, coordinated and carried out all the experiments and participated in manuscript preparation. GZ provided assistance for all experiments. All authors have read and approved the content of the manuscript.

## Pre-publication history

The pre-publication history for this paper can be accessed here:

http://www.biomedcentral.com/1471-2415/14/94/prepub

## Supplementary Material

Additional file 1: Table S1 Methodological quality (STROBE Statement-checklist) of included studies in the meta-analysis. Click here for file

## References

[B1] Thylefors B Negrel A Pararajasegaram R Dadzie K Global data on blindness Bull World Health Organ 1995 73 1 115 7704921 PMC2486591

[B2] JMJ R Leading causes of blindness worldwide Bull Soc Belge Ophtalmol 2002 283 19 25 12058483

[B3] Pollreisz A Schmidt-Erfurth U Diabetic cataract—pathogenesis, epidemiology and treatment J Ophthalmol 2010 2010 1 8 10.1155/2010/608751 PMC2903955 20634936

[B4] Janghorbani M Amini M Cataract in type 2 diabetes mellitus in Isfahan, Iran: incidence and risk factors Ophthalmic Epidemiol 2004 11 5 347 358 10.1080/09286580490888753 15590582

[B5] Jacques PF Moeller SM Hankinson SE Chylack LT Jr Rogers G Tung W Wolfe JK Willett WC Taylor A Weight status, abdominal adiposity, diabetes, and early age-related lens opacities Am J Clin Nutr 2003 78 3 400 405 12936921 10.1093/ajcn/78.3.400

[B6] Klein BE Klein R Wang Q Moss SE Older-onset diabetes and lens opacities. The Beaver Dam Eye Study Ophthalmic Epidemiol 1995 2 1 49 55 10.3109/09286589509071451 7585233

[B7] Olafsdottir E Andersson DK Stefansson E The prevalence of cataract in a population with and without type 2 diabetes mellitus Acta Ophthalmol 2012 90 4 334 340 10.1111/j.1755-3768.2011.02326.x 22176834

[B8] Machan CM Type 2 diabetes mellitus and the prevalence of age-related cataract in a clinic population 2012 Waterloo, Ontario, Canada: University of Waterloo

[B9] Chylack LT Wolfe JK Singer DM Leske MC Bullimore MA Bailey IL Friend J McCarthy D Wu S-Y The lens opacities classification system III Arch Ophthalmol 1993 111 1506 10.1001/archopht.1993.01090060119035 8512486

[B10] Von Elm E Altman DG Egger M Pocock SJ Gøtzsche PC Vandenbroucke JP The Strengthening the Reporting of Observational Studies in Epidemiology (STROBE) statement: guidelines for reporting observational studies Prev Med 2007 45 4 247 251 10.1016/j.ypmed.2007.08.012 17950122

[B11] Lau J Ioannidis JP Schmid CH Quantitative synthesis in systematic reviews Ann Intern Med 1997 127 9 820 826 10.7326/0003-4819-127-9-199711010-00008 9382404

[B12] Higgins JP Thompson SG Deeks JJ Altman DG Measuring inconsistency in meta-analyses BMJ 2003 327 7414 557 10.1136/bmj.327.7414.557 12958120 PMC192859

[B13] Begg CB Mazumdar M Operating characteristics of a rank correlation test for publication bias Biometrics 1994 50 4 1088 1101 10.2307/2533446 7786990

[B14] Egger M Davey Smith G Schneider M Minder C Bias in meta-analysis detected by a simple, graphical test BMJ (Clin Res Ed) 1997 315 7109 629 634 10.1136/bmj.315.7109.629 PMC2127453 9310563

[B15] Foster PJ Wong TY Machin D Johnson GJ Seah SK Risk factors for nuclear, cortical and posterior subcapsular cataracts in the Chinese population of Singapore: the Tanjong Pagar Survey Br J Ophthalmol 2003 87 9 1112 1120 10.1136/bjo.87.9.1112 12928278 PMC1771847

[B16] Leske MC Wu SY Hennis A Connell AM Hyman L Schachat A Diabetes, hypertension, and central obesity as cataract risk factors in a black population. The Barbados Eye Study Ophthalmology 1999 106 1 35 41 10.1016/S0161-6420(99)90003-9 9917778

[B17] Machan CM Hrynchak PK Irving EL Age-related cataract is associated with type 2 diabetes and statin use Optom Vis Sci 2012 89 8 1165 1171 10.1097/OPX.0b013e3182644cd1 22797512

[B18] Rotimi C Daniel H Zhou J Obisesan A Chen G Chen Y Amoah A Opoku V Acheampong J Agyenim-Boateng K Prevalence and determinants of diabetic retinopathy and cataracts in West African type 2 diabetes patients Ethn Dis 2003 13 2 Suppl 2 S110 S117 13677425

[B19] Tan JS Wang JJ Mitchell P Influence of diabetes and cardiovascular disease on the long-term incidence of cataract: the Blue Mountains eye study Ophthalmic Epidemiol 2008 15 5 317 327 10.1080/09286580802105806 18850468

[B20] Ivancic D Mandic Z Barac J Kopic M Cataract surgery and postoperative complications in diabetic patients Coll Antropol 2005 29 Suppl 1 55 58 16193678

[B21] Patel PM Jivani N Malaviya S Gohil T Bhalodia Y Cataract: A major secondary diabetic complication Int Curr Pharm 2012 1 7 180 185

[B22] Squirrell D Bhola R Bush J Winder S Talbot J A prospective, case controlled study of the natural history of diabetic retinopathy and maculopathy after uncomplicated phacoemulsification cataract surgery in patients with type 2 diabetes Br J Ophthalmol 2002 86 5 565 571 10.1136/bjo.86.5.565 11973256 PMC1771134

[B23] Klein BE Klein R Moss SE Incidence of cataract surgery in the Wisconsin Epidemiologic Study of Diabetic Retinopathy Am J Ophthalmol 1995 119 3 295 300 7872389 10.1016/s0002-9394(14)71170-5

[B24] Obrosova IG Chung SSM Kador PF Diabetic cataracts: mechanisms and management Diabetes Metab Res Rev 2010 26 3 172 180 10.1002/dmrr.1075 20474067

[B25] Onakpoya OH Bekibele CO Adegbehingbe SA Cataract surgical outcomes in diabetic patients: case control study Middle East Afr J Ophthalmol 2009 16 2 88 10.4103/0974-9233.53868 20142968 PMC2813591

[B26] Klein BE Klein R Lee KE Diabetes, cardiovascular disease, selected cardiovascular disease risk factors, and the 5-year incidence of age-related cataract and progression of lens opacities: the Beaver Dam Eye Study Am J Ophthalmol 1998 126 6 782 790 10.1016/S0002-9394(98)00280-3 9860001

[B27] Kyselova Z Stefek M Bauer V Pharmacological prevention of diabetic cataract J Diabetes Complicat 2004 18 2 129 140 10.1016/S1056-8727(03)00009-6 15120709

[B28] Lin H-J Huang Y-C Lin J-M Wu J-Y Chen L-A Lin C-J Tsui Y-P Chen C-P Tsai F-J Single-nucleotide polymorphisms in chromosome 3p14. 1-3p14. 2 are associated with susceptibility of Type 2 diabetes with cataract Mol Vis 2010 16 1206 20664687 PMC2901187

