# Supplementary material for: Meta-analysis of the risk of cataract in type 2 diabetes
Source: BMC Ophthalmol. 2014 Jul 24;14:94. doi: 10.1186/1471-2415-14-94 (PMC4113025; doi:10.1186/1471-2415-14-94)
Supplement: Additional file 1: Table S1 — Methodological quality (STROBE Statement-checklist) of included studies in the meta-analysis. [file 1471-2415-14-94-S1.doc]

Supplement Table 1 Methodological quality(STROBE Statement-checklist) of included studies in the meta-analysis.

| **Item No** | **Machan** | **Tan** | **Rotimi** | **Olafsdottir** | **Jacques** | **Klein** | **Leske** | **Foster** |
| --- | --- | --- | --- | --- | --- | --- | --- | --- |
| 1 | 1 | 1 | 1 | 1 | 1 | 1 | 1 | 1 |
| 2 | 1 | 1 | 1 | 1 | 1 | 1 | 1 | 1 |
| 3 | 1 | 1 | 1 | 1 | 1 | 1 | 1 | 1 |
| 4 | 1 | 1 | 1 | 1 | 1 | —— | 1 | 1 |
| 5 | 1 | 1 | 1 | 1 | 1 | 1 | 1 | 1 |
| 6 | 1 | 1 | 1 | 1 | 1 | 1 | 1 | 1 |
| 7 | 1 | 1 | 1 | 1 | 1 | 1 | 1 | 1 |
| 8 | 1 | 1 | 1 | 1 | 1 | 1 | 1 | 1 |
| 9 | 1 | 1 | 1 | 1 | 1 | 1 | 1 | 1 |
| 10 | —— | —— | —— | —— | —— | —— | —— | —— |
| 11 | 1 | 1 | 1 | 1 | 1 | 1 | 1 | 1 |
| 12 | 1 | 1 | 1 | 1 | 1 | 1 | 1 | 1 |
| 13 | 1 | 1 | 1 | 1 | 1 | 1 | —— | 1 |
| 14 | 1 | 1 | 1 | 1 | 1 | 1 | 1 | 1 |
| 15 | 1 | 1 | 1 | 1 | 1 | 1 | 1 | 1 |
| 16 | 1 | 1 | 1 | 1 | 1 | 1 | 1 | 1 |
| 17 | —— | 1 | —— | 1 | 1 | —— | —— | 1 |
| 18 | 1 | 1 | 1 | 1 | 1 | 1 | 1 | 1 |
| 19 | 1 | 1 | —— | —— | 1 | —— | —— | 1 |
| 20 | 1 | 1 | 1 | 1 | 1 | 1 | 1 | 1 |
| 21 | 1 | 1 | 1 | 1 | 1 | 1 | 1 | 1 |
| 22 | 1 | 1 | —— | 1 | 1 | —— | 1 | 1 |
| Total | 20 | 21 | 18 | 20 | 21 | 17 | 18 | 21 |
